# Genome-Wide Identification and Characterization of HSP90-RAR1-SGT1-Complex Members From *Arachis* Genomes and Their Responses to Biotic and Abiotic Stresses

**DOI:** 10.3389/fgene.2021.689669

**Published:** 2021-08-27

**Authors:** Cuiling Yuan, Chunjuan Li, Xiaobo Zhao, Caixia Yan, Juan Wang, Yifei Mou, Quanxi Sun, Shihua Shan

**Affiliations:** Shandong Peanut Research Institute, Qingdao, China

**Keywords:** HSP90-RAR1-SGT1 complex, molecular characteristics, stress-responsive, protein interaction, *Arachis*

## Abstract

The molecular chaperone complex HSP90-RAR1-SGT1 (HRS) plays important roles in both biotic and abiotic stress responses in plants. A previous study showed that wild peanut *Arachis diogoi SGT1* (*AdSGT1*) could enhance disease resistance in transgenic tobacco and peanut. However, no systematic analysis of the HRS complex in *Arachis* has been conducted to date. In this study, a comprehensive analysis of the HRS complex were performed in *Arachis*. Nineteen *HSP90*, two *RAR1* and six *SGT1* genes were identified from the allotetraploid peanut *Arachis hypogaea*, a number close to the sum of those from the two wild diploid peanut species *Arachis duranensis* and *Arachis ipaensis*. According to phylogenetic and chromosomal location analyses, thirteen orthologous gene pairs from *Arachis* were identified, all of which except *AhHSP90-A8*, *AhHSP90-B9*, *AdHSP90-9*, and *AiHSP90-9* were localized on the syntenic locus, and they shared similar exon-intron structures, conserved motifs and expression patterns. Phylogenetic analysis showed that HSP90 and RAR1 from dicot and monocot plants diverged into different clusters throughout their evolution. Chromosomal location analysis indicated that *AdSGT1* (the orthologous gene of *AhSGT1-B3* in this study) might provide resistance to leaf late spot disease dependent on the orthologous genes of *AhHSP90-B10* and *AhRAR1-B* in the wild peanut *A. diogoi*. Several HRS genes exhibited tissue-specific expression patterns, which may reflect the sites where they perform functions. By exploring published RNA-seq data, we found that several HSP90 genes play major roles in both biotic and abiotic stress responses, especially salt and drought responses. Autoactivation assays showed that AhSGT1-B1 could not be used as bait for yeast two-hybrid (Y2H) library screening. AhRAR1 and AhSGT1 could strongly interact with each other and interact with AhHSP90-B8. The present study represents the first systematic analysis of HRS complex genes in *Arachis* and provides valuable information for functional analyses of HRS complex genes. This study also offers potential stress-resistant genes for peanut improvement.

## Introduction

Peanut, also known as groundnut (*Arachis hypogaea* L.), is an economically important, nutritious and protein-rich oilseed crop species grown around the world (Zhuang et al., 2019). Abiotic stresses such as salt (Zhang et al., 2020), drought (Zhao et al., 2018), calcium deficiency (Chen et al., 2019) and biotic stresses such as *Aspergillus flavus* contamination (Zhao et al., 2020) strongly affect peanut quality and yield. Breeding stress-resistant peanut cultivars is an effective way to avoid declines in quality and yield. The mining and characterization of stress-responsive genes or gene families can facilitate crop quality improvement. However, few stress-responsive genes in peanut have been identified. In recent years, genome information (Bertioli et al., 2016; Zhuang et al., 2019) and many RNA-seq data (Clevenger et al., 2016; Zhao et al., 2018, 2020; Chen et al., 2019; Zhang et al., 2020) for peanut have become available, which are convenient for gene family characterization and mining stress-responsive genes.

Plants have evolved stress-resistance genes to cope with abiotic and biotic stresses. These genes include the three members of the HSP90-RAR1-SGT1 (HRS) complex, namely, HSP90 (heat shock protein 90), its chaperone RAR1 (required for MLA12 resistance) and SGT1 [suppressor of the G2 allele of SKP1 (S-phase kinase–associated protein 1)] (Shirasu, 2009). The HRS complex plays important roles in plant resistance to disease by stabilizing many NLR proteins. RAR1 was originally cloned from barley and encodes a protein containing two highly conserved zinc-binding domains, CHORD1 and CHORD2 (Shirasu et al., 1999). SGT1 was initially identified as involved in the yeast kinetochore assembly pathway (Krishna and Gloor, 2001) and contains five regions: a tetratricopeptide repeat (TPR) domain, two variable regions (VR1 and VR2), the CS (CHORD-containing protein and SGT1) domain, and the SGS (SGT1-specific motif) domain. The HSP90 family is a highly conserved molecular chaperone that plays diverse roles in plants (Krishna and Gloor, 2001; Kadota and Shirasu, 2011). It consists of three segments: an N-terminal ATPase domain (N), a substrate-binding domain in the middle (M), and a C-terminal dimerization domain (Kadota and Shirasu, 2011). HSP90, RAR1, and SGT1 can interact with each other, and their interaction is essential for their functions in biotic stress resistance (Shirasu, 2009; Ito et al., 2015). HRS-related genes are also involved in the abiotic stress response (Reddy et al., 2011; Shanmugam et al., 2016; Zhang et al., 2017; Vishwakarma et al., 2018). *E. coli* expressing *Pennisetum glaucum HSP90* exhibits enhanced tolerance to heat, salt and dehydration stresses (Reddy et al., 2011). Overexpression of the *rice Hsp90* gene can significantly increase the salt stress response of transgenic tobacco (Liu et al., 2006). The *BolSGT1* gene can potentially improve *B. oleracea* resistance to abiotic stresses such as heat, cold, and salt (Shanmugam et al., 2016). The expression of barley HSP90 genes has been shown to be upregulated under heat stress, heavy metal stress and calcium chloride treatment (Chaudhary et al., 2019). However, it seems that RAR1 is not involved in the abiotic stress response.

HRS complex-related genes have been identified in many plant species (Krishna and Gloor, 2001; Zhang et al., 2013, 2017; Agarwal et al., 2016; Song et al., 2019), for example, 7 in *Arabidopsis* (Krishna and Gloor, 2001) and 9 in rice (Hu et al., 2009). In common wheat, *TaHSP90* genes encode three types of HSP90 proteins (TaHSP90.1, TaHSP90.2, and TaHSP90.3) (Wang et al., 2011), and each TaHSP90 has three homologous genes in each genome (for example, TaHSP90.1-A1, TaHSP90.1-B1, and TaHSP90.1-C1). Five *HSP90* genes in chickpea, seven in pigeonpea, six in common bean, and five in *Medicago* and *Lotus* have been identified (Agarwal et al., 2016). RAR1 exists widely in plants but is absent in *Chlamydomonas* (Shirasu, 2009). Three distinct RAR1 homeologs (TaRAR1-A1, TaRAR1-B1, and TaRAR1-D1) have been isolated from common wheat (Wang et al., 2015). *Arabidopsis* contains two SGT1 isoforms, SGT1a and SGT1b (Austin et al., 2002). Three different SGT1 homeologs (TaSGT1-A1, TaSGT1-B1, and TaSGT1-D1) have been identified in common wheat (Wang et al., 2015).

The pathogen-induced SGT1 of wild peanut *Arachis diogoi* can induce cell death and enhance disease resistance in transgenic tobacco and peanut (Kumar and Kirti, 2015). However, the understanding of HRS-related genes in *Arachis* is still very poor. In this study, HRS-related genes were identified from both wild peanuts and cultivated peanuts. We also conducted chromosomal location, phylogenetic, gene structure, conserved motif, and tissue expression profiling analyses. In addition, expression profiling of HRS-related genes under abiotic and biotic stresses in cultivated peanut was performed. Furthermore, probable protein interactions among the *RAR1*, *SGT1*, and *HSP90* genes were investigated.

## Materials and Methods

### Identification of HRS-Related Proteins in *Arachis* Genomes

All HRS-related proteins were obtained from the PeanutBase database^1^ (Dash et al., 2016). These putative candidate proteins were manually verified using NCBI^2^ to confirm the presence of conserved motifs (CHORD1, CCCH, and CHORD2 for RAR1 proteins; TPR, CS, and SGS for SGT1 proteins; HATPase_c, HSP90 and MEEVD for HSP90 proteins). To verify the reliability of the search results, each protein sequence was examined using the domain analysis program SMART (Simple Modular Architecture Research Tool^3^) and the PFAM (Protein family) database^4^. Only the sequences containing these domains were retained. The protein isoelectric points (pIs) and molecular weights (MWs) were predicted using proteomics and sequence analysis tools on the ExPASy Proteomics Server^5^. The putative *Arabidopsis* orthologs for peanut HRS-related proteins were identified using a BLASTp search.

### Chromosomal Location, Gene Structure and Conserved Motif Analyses

The chromosomal location information of HRS-related proteins was obtained from the PeanutBase website (see text footnote 1) (Dash et al., 2016). The genes were mapped onto the chromosomes using the MapInspect software program^6^. The Gene Structure Display Server (GSDS) program (^7^ Hu et al., 2015) was used to elucidate the exon/intron organization of HRS-related genes. The Multiple Em for Motif Elicitation (MEME) program (^8^ Bailey et al., 2009) was used to illustrate the motifs in HRS-related protein sequences.

### Sequence Alignment and Phylogenetic Analyses

The representative *HSP90*, *RAR1*, and *SGT1* gene sequences from different species were retrieved from the NCBI database. Multiple alignment of their predicted amino acid sequences were performed using the computer program ClustalW (Larkin et al., 2007). Unrooted phylogenetic trees were constructed according to the neighbor-joining (NJ) method using MEGA 6.0 software (Tamura et al., 2013), and the bootstrap test was carried out with 1000 iterations.

### RNA-Seq-Based Expression Profiling of NAC Genes in Peanut

Gene expression data from 22 different tissues were obtained from Clevenger et al. (2016). To explore genes responsive to biotic and abiotic stresses, transcriptome sequencing information under salt, drought, low calcium, and *Aspergillus flavus* infection treatments was retrieved from the Short Read Archive at NCBI, with accession numbers SRR8177741 (Zhang et al., 2020), SRP093341 (Zhao et al., 2018), SRS3289849 (Chen et al., 2019), and PRJNA438019 (Zhao et al., 2020), respectively. The FPKM data or fold-change values for each HRS gene were log2 transformed and displayed in the form of heatmaps via HemI (Deng et al., 2014).

### Peanut Materials and Growth Conditions

Huayu 9303, a peanut cultivar with high yield and good quality, was grown in a temperature-controlled chamber at 20°C with a photoperiod of 16 h light and 8 h dark unless otherwise stated. After approximately 1 month, the leaves were collected, frozen immediately in liquid nitrogen and stored at −80°C.

### Gene Cloning and Plasmid Construction

Several HRS-related genes from the cultivated peanut Huayu 9303 were isolated and then subcloned them into plasmids for yeast two-hybrid (Y2H) assays. Total RNA was isolated using TRIzol reagent (Invitrogen). To clone the full-length coding regions of these genes, reverse transcription polymerase chain reaction (RT-PCR) was performed according to the M-MLV Reverse Transcriptase (Invitrogen) protocol. The amplification of all genes was performed with an initial denaturation at 98°C for 30 s, followed by 30 cycles at 98°C for 10 s, 58°C for 10 s, and 72°C for 1 min, and a final extension at 72°C for 10 min. PCR amplifications were performed using Phusion High Fidelity DNA polymerase (New England Biolabs). PCR products were first cloned into the pENTR/D TOPO vector (Invitrogen) and sequenced. The cloned genes were then recombined into the Gateway-compatible pGADT7 (AD) and pGBKT7 (BD) vectors for Y2H analysis.

### Y2H Analysis

For Y2H autoactivation and pairwise interaction assays, we implemented the Gal4-based Matchmaker Gold Yeast Two-Hybrid System (Clontech^9^). This system includes four independent reporters that allow testing interactions at different stringencies, with the use of a double dropout medium (DDO: SD/-Trp/-Leu), triple dropout medium (TDO: SD/-Trp/-Leu/-His), and quadruple dropout medium (QDO: SD/-Trp/-Leu/-His/-Ade) (Clontech). The plasmids BD-wXb12 and AD-wXb12IP2 were used as positive controls, and the interaction between BD-wXb12 and AD-wRAR1 served as a negative control (Pei et al., 2015).

## Results

### Identification and Characterization of HSP90 Genes

**FIGURE 1:**
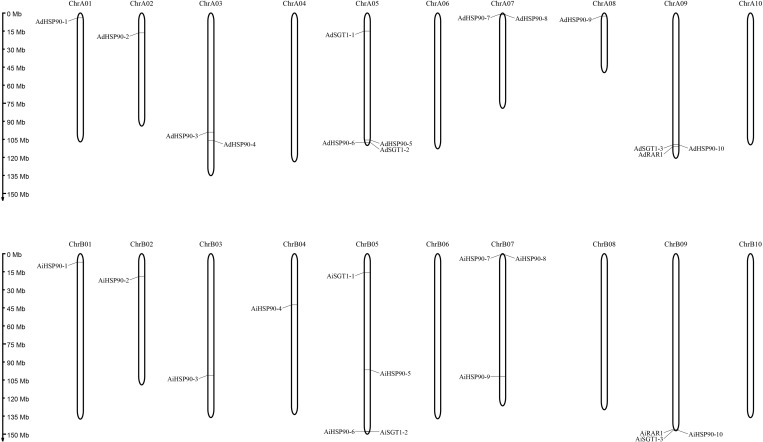
Chromosomal locations of HRS-related genes in the *A. duranensis* and *A. ipaensis* genomes. The chromosome numbers are shown at the top of each chromosome (black bars). The left side of each chromosome corresponds to the approximate location of each HRS-related gene.

Ten *HSP90* genes were identified from the genomes of the diploid wild peanut species *A. duranensis* and *A. ipaensis*. Because of the lack of a nomenclatural method for HSP90 genes, we named these genes *AdHSP90-1* to *AdHSP90-10* and *AiHSP90-1* to *AiHSP90-10* according to their arrangement on the chromosome (Figure 1). The *HSP90* genes identified in this study had MWs ranging from 73.60 to 92.41 kDa. The encoded proteins varied from 646 amino acids (aa) to 815 aa in length, with an average of 732.9 aa. The pIs of the predicted proteins were determined as shown in Table 1. In the allotetraploid cultivated peanut *A. hypogaea*, 9 and 10 *HSP90* genes were localized on the A and B subgenomes, respectively. We named them *AhHSP90-A1* to *AhHSP90-A9* and *AhHSP90-B1* to *AhHSP90-B10*. One *HSP90* gene was lost in the A subgenome of cultivated peanut. The size of the HSP90 proteins in *A. hypogaea* ranged from 340 to 812 aa, with an average of 711.8 aa (Table 1).

**TABLE 1:** The HRS-related genes in *Arachis* genomes.

Gene	Gene ID	Gene model name	Gene location	Length (aa)	MW (kDa)	pI
HSP90	AdHSP90-1	Aradu.K62H7	Chr01:4042591…4045539	733	83.87	5.06
	AdHSP90-2	Aradu.U2NBF	Chr02:16444152…16453651	646	73.60	4.92
	AdHSP90-3	Aradu.QQ0H9	Chr03:99109508…99111882	705	81.51	5.11
	AdHSP90-4	Aradu.5WK97	Chr03:105917267…105926473	815	90.50	7.68
	AdHSP90-5	Aradu.EYD2G	Chr05:105368002…105374644	786	88.97	4.94
	AdHSP90-6	Aradu.JL6EF	Chr05:107365559…107368461	705	80.90	4.96
	AdHSP90-7	Aradu.42GMX	Chr07:1034064…1039317	799	90.93	5.37
	AdHSP90-8	Aradu.SF8G0	Chr07:1367922…1370709	699	80.19	5.00
	AdHSP90-9	Aradu.YNP6V	Chr08:2522243…2527662	807	92.41	4.93
	AdHSP90-10	Aradu.9Z8WQ	Chr09:109313308…109315726	704	81.08	5.05
	AiHSP90-1	Araip.JZ997	Chr01:7176588…7179545	731	83.67	5.04
	AiHSP90-2	Araip.XZ287	Chr02:19158695…19166049	706	80.86	5.12
	AiHSP90-3	Araip.MQB8Q	Chr03:100895556…100897929	705	81.50	5.04
	AiHSP90-4	Araip.L8W42	Chr04:42496399…42497955	691	79.33	5.22
	AiHSP90-5	Araip.H6BYJ	Chr05:96289302…96296066	800	90.66	4.96
	AiHSP90-6	Araip.SBH16	Chr05:147726485…147729369	704	80.76	4.98
	AiHSP90-7	Araip.1550X	Chr07:763865…769158	795	90.44	5.41
	AiHSP90-8	Araip.H4QT2	Chr07:1064695…1067477	699	80.19	5.00
	AiHSP90-9	Araip.H5GIN	Chr07:102214153…102220288	762	87.29	5.01
	AiHSP90-10	Araip.P7YCF	Chr09:146848097…146850461	666	76.63	5.04
	AhHSP90-A1	Arahy.GX1P2J	Chr01:4281968…4285697	699	80.06	4.99
	AhHSP90-A2	Arahy.V8HRRV	Chr02:17799292…17804557	744	84.64	5.19
	AhHSP90-A3	Arahy.173LZF	Chr03:107511725…107516028	705	81.50	5.08
	AhHSP90-A4	Arahy.E9M6IW	Chr05:111511442…111518352	800	90.69	4.96
	AhHSP90-A5	Arahy.H9FJ3E	Chr05:113647397…113650729	704	80.79	4.98
	AhHSP90-A6	Arahy.LFBK1H	Chr07:289959…300481	799	90.87	5.41
	AhHSP90-A7	Arahy.B8WNJH	Chr07:603124…606501	701	80.45	4.98
	AhHSP90-A8	Arahy.T76A9L	Chr08:2447294…2452920	805	92.24	4.94
	AhHSP90-A9	Arahy.I9LWH3	Chr09:108774713…108778357	704	81.14	5.03
	AhHSP90-B1	Arahy.Q1M4CJ	Chr11:7521403…7525135	699	80.09	4.99
	AhHSP90-B2	Arahy.JLML9R	Chr12:20661661…20667084	809	91.65	5.57
	AhHSP90-B3	Arahy.L5T5KA	Chr13:109991247…109995578	705	81.50	5.04
	AhHSP90-B4	Arahy.8JE878	Chr15:27695376…27698350	340	50.09	5.39
	AhHSP90-B5	Arahy.TY031Z	Chr15:104239495…104246396	800	90.66	4.96
	AhHSP90-B6	Arahy.YQG5EZ	Chr15:158652587…158655685	687	78.74	4.95
	AhHSP90-B7	Arahy.LY71N7	Chr17:1627246…1637029	812	92.38	5.49
	AhHSP90-B8	Arahy.H890L5	Chr17:1942821…1946198	699	80.20	5.00
	AhHSP90-B9	Arahy.P8FSM4	Chr17:109413805…109419947	763	87.38	4.92
	AhHSP90-B10	Arahy.BDFQ12	Chr19:158448247…158451755	550	63.85	5.34
RAR1	AdRAR1	Aradu.1U59X	Chr09:110821126…110823942	277	30.57	6.86
	AiRAR1	Araip.W0D6Y	Chr09:146128854…146131867	271	29.70	8.26
	AhRAR1-A	Arahy.939HGU	Chr09:110273928…110277287	223	24.48	6.81
	AhRAR1-B	Arahy.ATT3CU	Chr19:157690846…157694117	223	24.46	7.11
SGT1	AdSGT1-1	Aradu.XZ2EW	Chr05:15006013…15009310	371	41.75	5.37
	AdSGT1-2	Aradu.KZZ75	Chr05:107486599…107491066	303	33.69	4.90
	AdSGT1-3	Aradu.IR41I	Chr09:109252282…109254862	361	40.53	5.81
	AiSGT1-1	Araip.0178A	Chr05:15727576…15731506	371	41.85	5.37
	AiSGT1-2	Araip.U037H	Chr05:147870552…147874956	318	35.81	5.37
	AiSGT1-3	Araip.LE6XA	Chr09:146881056…146883664	361	40.43	5.81
	AhSGT1-A1	Arahy.K7FQ7Q	Chr05:41476362…41480242	371	41.78	5.37
	AhSGT1-A2	Arahy.A992JM	Chr05:113798861…113803312	346	38.78	5.02
	AhSGT1-A3	Arahy.DRAR7X	Chr09:108723555…108726162	366	41.00	6.00
	AhSGT1-B1	Arahy.MC01IH	Chr15:16242613…16246474	371	41.85	5.37
	AhSGT1-B2	Arahy.4FT00Z	Chr15:158809356…158813745	361	41.18	8.69
	AhSGT1-B3	Arahy.MU20M7	Chr19:158488297…158490908	361	40.43	5.81

**FIGURE 2:**
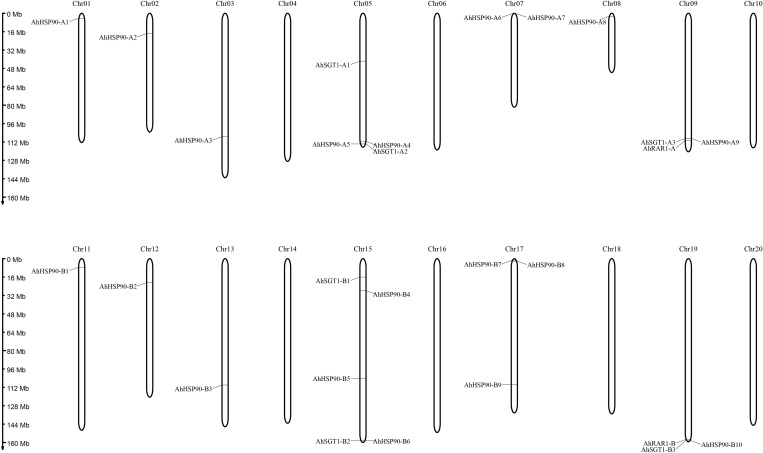
Chromosomal locations of HRS-related genes in the *A. hypogaea* genome. The chromosome numbers are shown at the top of each chromosome (black bars). The left side of each chromosome corresponds to the approximate location of each HRS-related gene.

**FIGURE 3:**
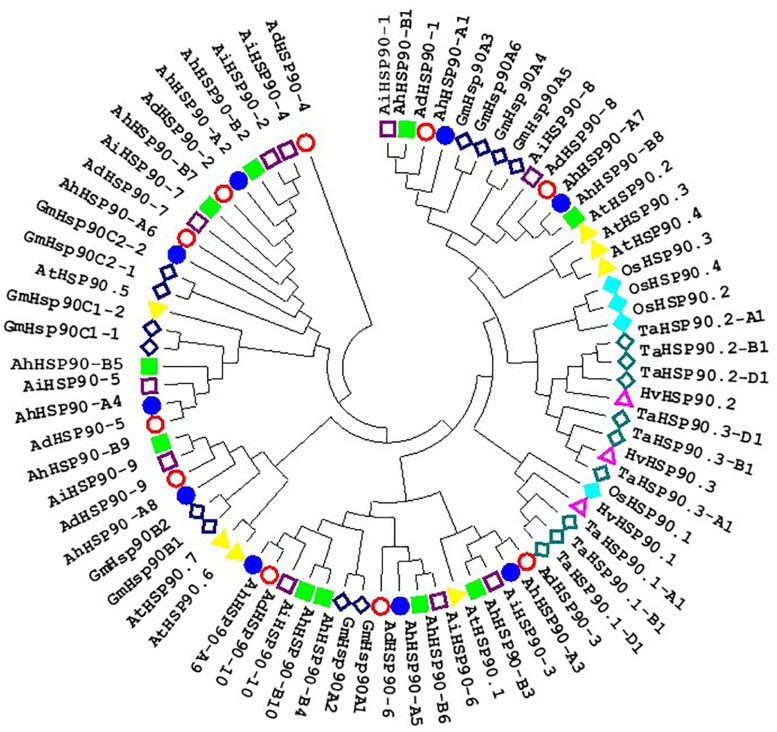
Phylogenetic tree of HSP90 proteins of *Arachis*, *Arabidopsis*, rice, soybean, barley, and wheat. Multiple sequence alignment of peanut NAC proteins was performed using ClustalW, and the phylogenetic tree was constructed using MEGA 6.0 by the neighbor-joining method with 1000 bootstrap replicates.

**FIGURE 4:**
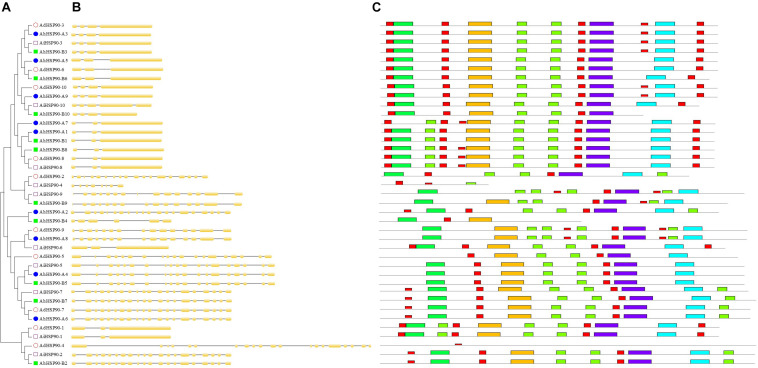
Phylogenetic analysis, gene structures, and conserved motifs of *Arachis* HSP90 genes. **(A)** Phylogenetic tree of *Arachis* HSP90s. **(B)** Exon-intron structure of *Arachis* HSP90 genes. **(C)** Ten conserved motifs in HSP90 proteins.

Chromosomal location analysis showed that all 39 HSP90 genes were randomly distributed on every chromosome except chromosomes A4, A6, A10, B6, B8, and B10 of *A. duranensis* and *A. ipaensis* (Figure 1) and chromosomes 4, 6, 10, 14, 16, 18, and 20 of cultivated peanut (Figure 2). Orthologous gene pairs (Table 2) were obtained according to the phylogenetic analysis results (Figure 3). In general, orthologous genes were located on the syntenic locus of each chromosome. *AdHSP90-9* and *AhHSP90-A8* localized on chromosome A8 of *A. duranensis* and chromosome 7 of *A. hypogaea*, while their orthologous genes *AiHSP90-9* and *AhHSP90-B9* were assigned to chromosomes B8 and 17, respectively. The orthologous gene pairs from the same genome of wild and cultivated peanut (for example, *A. duranensis* and the A subgenome of *A. hypogaea*) usually shared high sequence similarity (Table 2). Some orthologous genes shared identical protein sequences; for example, AdHSP90-1 and AhHSP90-A1 shared the same amino acid sequence; however, their CDSs were slightly different. Most orthologous genes shared similar exon-intron structures and conserved motifs, for example, AdHSP90-3, AiHSP90-3, AhHSP90-A3, and AhHSP90-B3 (Figure 4). Most HSP90 genes contained 3 or 4 exons, while some had more than 19 exons, which might explain the diversity of HSP90 functions. Remarkably, *AdHSP90-4* contained 20 exons; however, there was only one motif in its putative protein sequence.

**TABLE 2:** Orthologous gene pairs in *Arachis* genomes.

*A. duranensis*	*A. ipaensis*	*A. hypogaea* A sub-genome	*A. hypogaea* B sub-genome	Chromosomal location	CDS identity (%)	Amino acid identity (%)
AdHSP90-1	AiHSP90-1	AhHSP90-A1	AhHSP90-B1	A1-B1-1-11	99.57	99.93
AdHSP90-2	AiHSP90-2	AhHSP90-A2	AhHSP90-B2	A2-B2-2-12	94.98	92.63
AdHSP90-3	AiHSP90-3	AhHSP90-A3	AhHSP90-B3	A3-B3-3-13	99.46	99.40
AdHSP90-5	AiHSP90-5	AhHSP90-A4	AhHSP90-B5	A5-B5-5-15	99.05	99.00
AdHSP90-6	AiHSP90-6	AhHSP90-A5	AhHSP90-B6	A5-B5-5-15	98.69	99.08
AdHSP90-7	AiHSP90-7	AhHSP90-A6	AhHSP90-B7	A7-B7-7-17	97.48	97.61
AdHSP90-8	AiHSP90-8	AhHSP90-A7	AhHSP90-B8	A7-B7-7-17	99.24	99.64
AdHSP90-9	AiHSP90-9	AhHSP90-A8	AhHSP90-B9	A8-B7-8-17	96.49	95.57
AdHSP90-10	AiHSP90-10	AhHSP90-A9	AhHSP90-B10	A9-B9-9-19	88.89	87.71
AdRAR1	AiRAR1	AhRAR1-A	AhRAR1-B	A9-B9-9-19	82.65	84.93
AdSGT1-1	AiSGT1-1	AhSGT1-A1	AhSGT1-B1	A5-B5-5-15	99.51	99.53
AdSGT1-2	AiSGT1-2	AhSGT1-A2	AhSGT1-B2	A5-B5-5-15	90.19	85.30
AdSGT1-3	AiSGT1-3	AhSGT1-A3	AhSGT1-B3	A9-B9-9-19	98.14	97.88

### Identification and Characterization of RAR1 Genes

**FIGURE 5:**
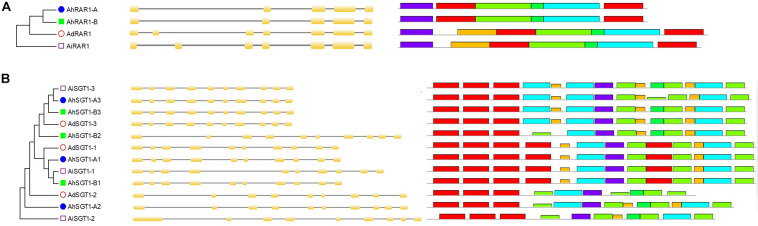
Phylogenetic analysis, gene structures, and conserved motifs of the *Arachis* RAR1 and SGT1 genes. **(A)** *Arachis* RAR1 genes; **(B)** *Arachis* SGT1 genes.

**FIGURE 6:**
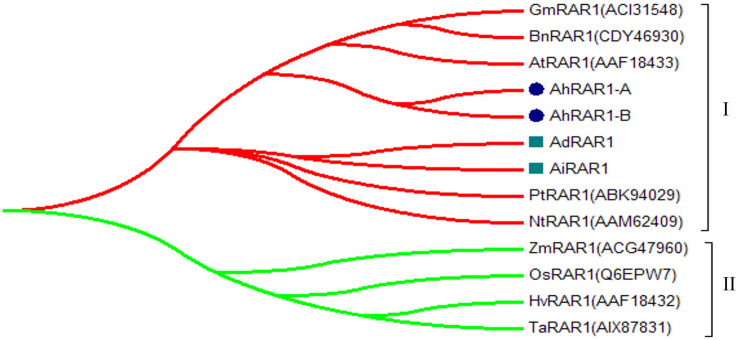
Phylogenetic tree of RAR1 genes from *Arachis*, *Arabidopsis*, soybean, barley, common wheat, rice, maize, tobacco, rape, and *Populus trichocarpa*.

Only one RAR1 gene was identified from each diploid, *A. duranensis* and *A. ipaensis*, and was named *AdRAR1* and *AiRAR1*, respectively. The deduced protein sequences of *AdRAR1* and *AiRAR1* contained 277 and 271 amino acid residues, respectively (Table 1). The *AdRAR1* and *AiRAR1* genes were assigned to the ends of chromosomes A9 and B9, respectively (Figure 1). The pIs of the predicted proteins of *AdRAR1* and *AiRAR1* were 6.86 and 8.26, respectively, and the MWs were 30.57 and 29.70 kDa, respectively (Table 1). Bioinformatics analyses indicated that there are two *RAR1* homologs in the allotetraploid cultivated peanut *A. hypogaea*. Not surprisingly, two *RAR1* genes were isolated from the cultivated peanut genome and named *AhRAR1-A* and *AhRAR1-B*. Both deduced amino acid sequences had a molecular weight of 24.5 kDa, and their theoretical pIs were 6.81 and 7.11. There were 5 exons in the AhRAR1 sequence and 7 exons in the wild peanut RAR1 sequence (Figure 5A). The *RAR1* genes were PCR-amplified from cultivated peanut to analyze their sequences and perform subsequent work. AhRAR1-A and AhRAR1-B shared a similarity of 98.66%, with 3 amino acid differences (Supplementary Figure 1). The encoded proteins were both 224 aa in length, different from the RAR1 proteins in wild peanut. The sequence comparison of RAR1 from cultivated peanut (*AhRAR1-A* and *AhRAR1-B*) and the model plant *Arabidopsis* (*AtRAR1*) revealed the same gene structure, as shown in Supplementary Figure 2. Both *AhRAR1-A* and *AtRAR1* consisted of 6 exons and 5 introns. Notably, *AhRAR1* consisted of only 3 base pairs in exon 2 (Supplementary Figures 2A,B). The deduced amino acid of *AhRAR1* was composed of three conserved domains: CHORD-I, CHORD-II, and a highly conserved CCCH domain between the CHORD domains (Supplementary Figure 2C). A phylogenetic tree was constructed based on the RAR1 protein sequence from diverse plant species (Figure 6). The results showed that monocot and dicot RAR1 proteins were separated into two distinct classes (I and II), and the RAR1s from cultivated and wild peanut were distributed among different branches. The phylogenetic analysis results together with the high similarity of amino acid sequences and the same chromosome locations indicated that *AdRAR1*, *AiRAR1*, *AhRAR1-A1*, and *AhRAR1-B1* were orthologous genes.

### Identification and Characterization of SGT1 Genes

We predicted 3 *SGT1* genes from the *A. duranensis* and *A. ipaensis* genomes. They were designated as *AdSGT1* to *AdSGT3* and *AiSGT1* to *AiSGT3* (Table 1). The *SGT1* genes identified in *A. duranensis* and *A. ipaensis* were located on chromosomes 5 and 9, encoded proteins ranging from 303 to 371 amino acid residues in length, and had the highest sequence similarity to the *Arabidopsis SGT1* gene (AAL33611.1). The pI of the predicted proteins ranged from 4.90 to 5.81. Six SGT1 genes were identified from the cultivated peanut genome. We named them *AhSGT1-A1* to *AhSGT1-A3* and *AhSGT1-B1* to *AhSGT1-B3*. They had MWs ranging from 40.24 to 41.85 kDa. The encoded proteins varied from 357 to 371 aa in length, with an average of 361 aa (Table 1). Amino acid multiple alignment of the SGT1s from peanut indicated that the *AdSGT1-1* and *AiSGT1-1* genes were orthologous to *AhSGT1-A1* and *AhSGT1-B1* (98.61% amino acid identity), and only a few amino acids differed between these genes (Supplementary Figure 3), which were located in the syntenic chromosome location (Figures 1, 2). The *AdSGT1-2* and *AdSGT1-3* genes were orthologs to *AiSGT1-2* and *AiSGT1-3*, and all were assigned to chromosome 5 (Figure 1). A phylogenetic tree was constructed based on the SGT1 protein sequences from both wild and cultivated peanut. Based on the chromosomal location and phylogenetic analysis results (Figures 1, 5B), this study deduced that *AhSGT1-A1* and *AhSGT1-B1* were orthologous to *AdSGT1-1* and *AiSGT1-1*, respectively. *AhSGT1-A2* and *AhSGT1-B2* were orthologous to *AdSGT1-2* and *AiSGT1-2*, and *AhSGT1-B2* had the same sequence as *AiSGT1-2*. *AhSGT1-A3* and AhSGT1*-B3* were orthologous to *AdSGT1-3* and *AiSGT1-3* and the previously reported *AdSGT1* from *A. diogoi* (Supplementary Figure 4). These orthologous genes shared similar exon-intron structures and conserved motifs (Figure 5B). *AdSGT1-1*, *AdSGT1-3* and their orthologs all consisted of 10 exons, while *AdSGT1-2* and its orthologs contained only 9 exons.

### Expression Profiling and Responses to Biotic and Abiotic Stresses

**FIGURE 7:**
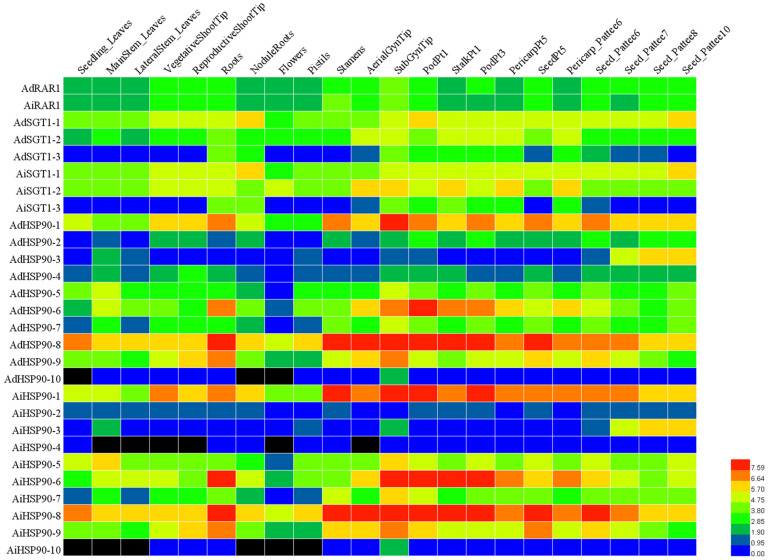
Expression profiling of HRS genes in 22 different tissues of *A. duranensis* and *A. ipaensis*. The FPKM data of each gene were retrieved from Clevenger et al. (2016), log2-transformed and displayed in the form of a heatmap by HemI. The color scale in the lower right indicates relative expression level: green represents a low level, and red indicates a high level. The 22 different tissues are listed at the top of the heat map. The HRS genes are listed on the left of the heatmap.

**FIGURE 8:**
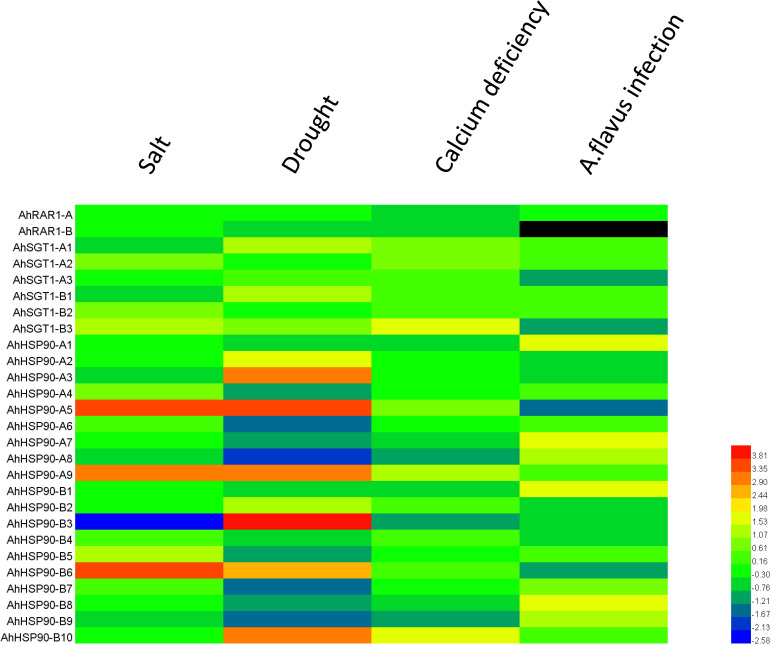
Transcriptome analysis of HRS genes in response to different stresses. The FPKM data of each gene under salt, drought, calcium deficiency, and *Aspergillus flavus* infection were sourced from previous studies (Zhao et al., 2018, 2020; Chen et al., 2019; Zhang et al., 2020). The fold change in HRS gene expression under stress treatment compared to that under control treatment was normalized, log2-transformed and displayed in the form of a heatmap by HemI. The color scale in the lower right indicates relative expression level: green represents a low level, and red indicates a high level. The 22 different tissues are listed at the top of the heat map. The HRS genes are listed on the left of the heatmap. The color scale in the lower right indicates relative expression level: green represents a low level, and red indicates a high level. Different stress treatments are shown along the top of the heat map. The HRS genes are listed on the left of the heatmap.

To understand the expression patterns of HRS-related genes, their expression levels in 22 different tissues or developmental stages were examined based on RNA-seq data (Clevenger et al., 2016). We found that orthologous genes exhibited similar expression patterns (for instance, *AdHSP90-1* and *AiHSP90-1*, *AdSGT1* and their orthologs in *A. ipaensis*). Notably, *AdRAR1* from *A. duranensis* and *AiRAR1* from *A. ipaensis* exhibited the same expression patterns and were constitutively expressed at similar levels in all 22 tissues or developmental stages (Figure 7). *AdHSP90-8* and *AiHSP90-8* were also expressed in 22 tissues and exhibited higher expression in every tissue than other HRS-related genes, while the expression of *AdHSP90-10* and *AiHSP90-10* was hardly detected in any tissue. Some genes exhibited tissue-specific expression patterns; for example, *AdHSP90-3* and its ortholog *AiHSP90-3* exhibited especially high expression in the later seed development stage. Eight genes (*AdHSP90-1*, *AdHSP90-6*, *AdHSP90-8*, and *AdHSP90-9* and their orthologs *AiHSP90-1*, *AiHSP90-6*, *AiHSP90-8*, and *AiHSP90-9*) were expressed at higher levels than other genes in roots. To identify genes involved in biotic and abiotic stresses, we comparatively reanalyzed RNA-seq data involved in the responses to salt (Zhang et al., 2020), drought (Zhao et al., 2018), low calcium (Chen et al., 2019), and *A. flavus* infection (Zhao et al., 2020). The results suggested that several HSP90 genes play important roles in salt- and drought-stress responses (Figure 8). *AhHSP90-A5*, *AhHSP90-A9* and *AhHSP90-B6* were significantly upregulated under salt stress, while *AhHSP90-B3* was significantly downregulated. Under drought treatment, *AhHSP90-A3*, *AhHSP90-A5*, *AhHSP90-A9*, *AhHSP90-B3, AhHSP90-B6*, and *AhHSP90-B10* were significantly upregulated, and *AhHSP90-A6*, *AhHSP90-A8*, and *AhHSP90-B9* were significantly downregulated. *AhSGT1-B3* was upregulated after salt treatment, while *AhSGT1-A1* and *AhSGT1-B1* were downregulated. Under drought stress, *AhSGT1-A1* and *AhSGT1-B1* were both upregulated. Under calcium-deficient conditions, *AhSGT1-B3*, *AhHSP90-A9*, and *AhHSP90-B10* exhibited higher expression levels than they did under calcium-sufficient conditions, while *AhRAR1*, *AhHSP90-A1*, *AhHSP90-A7*, *AhHSP90-A8*, *AhHSP90-B3*, and *AhHSP90-B9* exhibited the opposite tendency. The expression levels of *AhHSP90-A1*, *AhHSP90-A7*, and *AhHSP90-A8* and their orthologous genes *AhHSP90-B1*, *AhHSP90-B7*, and *AhHSP90-B8* were higher during *Aspergillus flavus* infection than under the control conditions. *AhSGT-A3*, *AhHSP90-A2*, *AhHSP90-A3*, and *AhHSP90-A5* and their orthologous genes *AhSGT-B3*, *AhHSP90-B2*, *AhHSP90-B3*, and *AhHSP90-B6* were downregulated after *Aspergillus flavus* infection (Figure 8). These orthologous genes exhibited the same expression patterns under stress conditions.

### Cloning of RAR1, SGT1, and HSP90 and the Interactions of These Proteins

**FIGURE 9:**
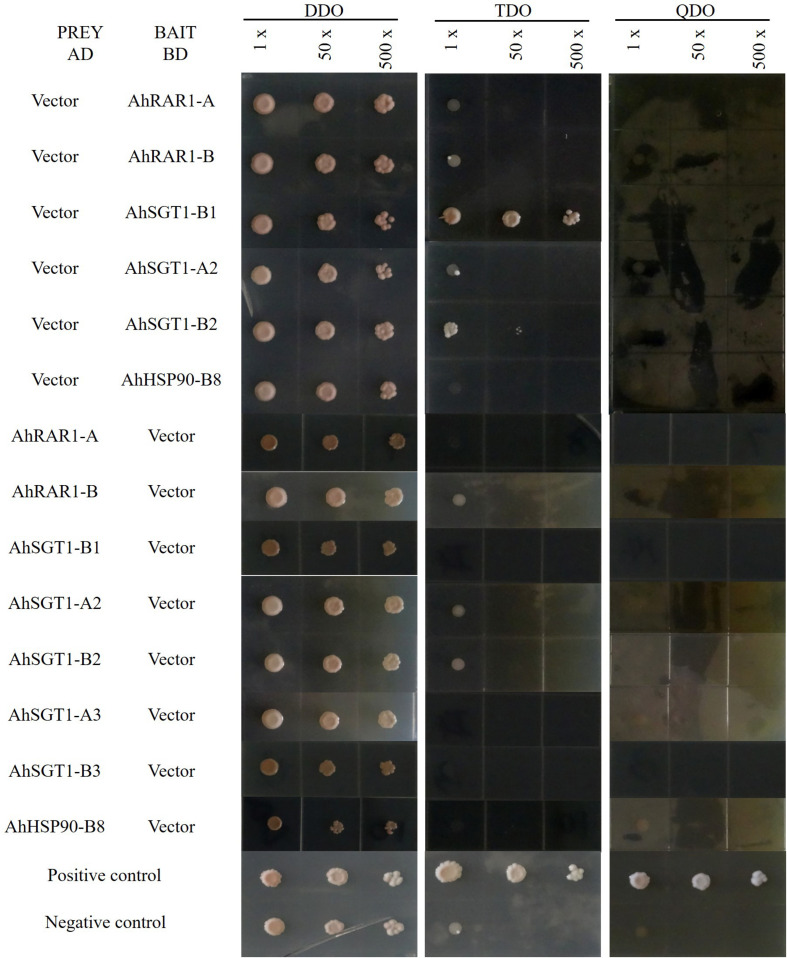
Autoactivation test of HRS proteins. The successfully cotransformed yeast containing both AD and BD plasmids was collected from DDO medium and inoculated on TDO and QDO media with the original resuspension (1×), a 50-fold dilution (50×), or a 500-fold dilution (500×).

**FIGURE 10:**
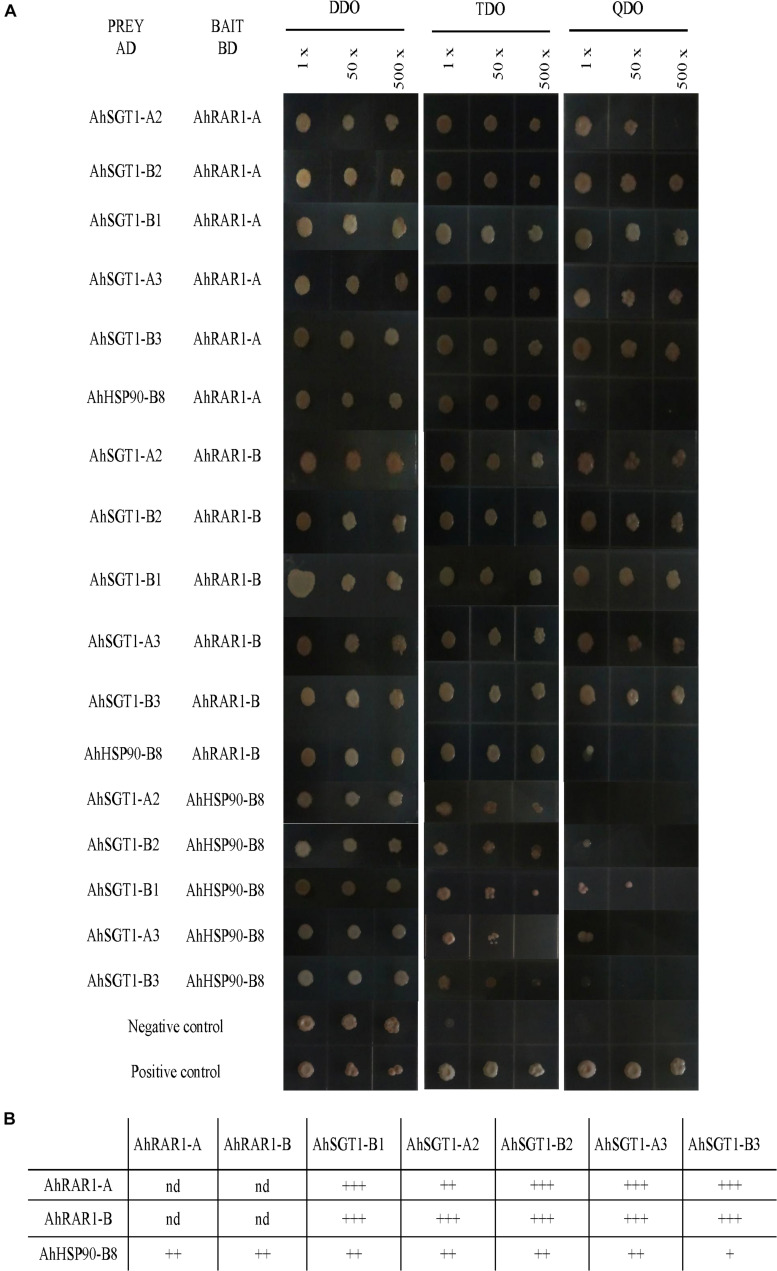
Pairwise interactions among HRS proteins. **(A)** Positive interactions were identified based on yeast growth in TDO and/or QDO media. The yeast inoculums were progressively diluted and used as the original resuspension (1×), 50-fold dilution (50×), or 500-fold dilution (500×). **(B)** Schematic representation of the HRS complex in peanut. The number following “+” represents the strength of the protein-protein interaction. nd: not detected.

To characterize their interactions, *AhRAR1-A*, *AhRAR1-B*, *AhSGT1-B1*, *AhSGT1-A2*, *AhSGT1-B2*, *AhSGT1-A3*, *AhSGT1-B3*, and *AhHSP90-B8* were isolated from the cultivated peanut Huayu 9303 and recombined them into AD and BD plasmids for Y2H analysis using the Gal4 System. These 8 genes, when used as prey and bait proteins, showed no or insignificant autoactivation on TDO and QDO media, with the exception of the AhSGT1-B1 bait on TDO medium (Figure 9). We then tested all possible pairwise interactions among AhRAR1-A, AhRAR1-B, AhSGT1-B1, AhSGT1-A2, AhSGT1-B2, AhSGT1-A3, AhSGT1-B3, and AhHSP90-B8 (Figure 10). AhHSP90-B8 interacted with AhRAR1s and AhSGT1s in the low stringency medium (TDO) but not in the QDO medium. Strong interaction between AhRAR1s and AhSGT1s were detected, as reflected by yeast growth on the high-stringency medium (QDO) (Figure 10).

## Discussion

HSP90 and its cochaperones RAR1 and SGT1 are usually involved in biotic and abiotic stress responses (Shirasu, 2009; Kadota and Shirasu, 2011). These gene families have been analyzed in several plants, such as *Arabidopsis* (Krishna and Gloor, 2001), common wheat (Wang et al., 2011, 2015), tobacco (Song et al., 2019), *Populus* (Zhang et al., 2013), chickpea and pigeonpea (Agarwal et al., 2016). A previous study showed that an *SGT1* gene from the wild peanut *A. diogoi* (*AdSGT1*) could enhance disease resistance in transgenic tobacco and peanut (Kumar and Kirti, 2015). However, it is still unknown whether HSP90 and RAR1 are expressed in *Arachis* species. In this study, a systemic analysis of HRS genes in both wild peanut and cultivated peanut were performed.

### Identification and Characterization of HRS Genes in *Arachis* Species

Ten, 10, and 19 HSP90 genes were retrieved from the genomes of the diploid wild peanut *A. duranensis*, the diploid wild peanut *A. ipaensis* and the allotetraploid cultivated peanut *A. hypogaea*, respectively (Table 1). One HSP90 gene was lost in the A subgenome of the allotetraploid cultivated peanut genome, which possibly attributed from incorrect splicing or genome sequencing errors. The number of HSP90 genes in cultivated peanut was nearly the sum of that of the two wild peanuts. Similar results were observed for the RAR1 and SGT1 genes, with two RAR1 and six SGT1 genes detected in cultivated peanut and only one RAR1 and three SGT1 genes identified in wild peanut (Table 1). This phenomenon could be attributed to the doubling of chromosomes in diploid wild species during the formation of tetraploid cultivated species (Bertioli et al., 2016; Zhuang et al., 2019). A similar finding was obtained in common wheat, in which the HRS complex has been well studied. There are 3 HSP90 genes in each chromosome of common wheat, that is, nine HSP90 genes in heterohexaploid wheat (AABBDD) (Wang et al., 2011), but only three HSP90 genes in its relative species diploid barley (Pei et al., 2015). There is one RAR1 gene and one SGT1 gene in the barley genome (Pei et al., 2015) and 3 RAR1 and 3 SGT1 genes in the wheat genome (Wang et al., 2011). In plants, the number of HSP90 genes varies greatly. Here, there were nineteen HSP90 genes in the cultivated peanut genome, which is more than the number reported in barely (3) (Pei et al., 2015), wheat (9) (Wang et al., 2011), *Arabidopsis* (7) (Krishna and Gloor, 2001), rice (9) (Hu et al., 2009), chickpea (5) (Agarwal et al., 2016), pigeonpea (11) (Agarwal et al., 2016), common bean (6) (Agarwal et al., 2016), *Medicago* (5) (Agarwal et al., 2016), *Lotus* (5) (Agarwal et al., 2016), and tobacco (11) (Song et al., 2019). This difference may be attributed to cultivated peanut being an allotetraploid plant in which genes have been replicated. These genes share similar intron/exon structures, conserved motifs, and high collinearity chromosomal locations and are considered orthologous genes (Song et al., 2016; Altenhoff et al., 2019). Thirteen orthologous gene pairs in the *Arachis* genomes were detected in this study (Table 2), for example, *AdHSP90-3*, *AiHSP90-3*, *AhHSP90-A3* and *AhHSP90-B3* (Figures 3, 4). Phylogenetic analysis showed that the orthologous genes tended to form clusters and shared high sequence similarity (Table 2), which indicated that these orthologous genes might perform the same functions. For example, we speculate that the function of *AdHSP90-3*, *AiHSP90-3*, *AhHSP90-A3*, and *AhHSP90-B3* is the same as that of *AtHSP90.1*, which is essential for both abiotic and biotic stress responses in *Arabidopsis* (Krishna and Gloor, 2001; Haralampidis et al., 2002; Takahashi et al., 2003). Most of the orthologous genes were located on the syntenic locus of each chromosome. The different chromosome locations of orthologous genes *AdHSP90-9*, *AhHSP90-A8*, *AiHSP90-9*, and *AhHSP90-B9* suggested that gene locus changes in chromosome locations occurred after divergence and duplication during *Arachis* evolution. This study found that in the phylogenetic tree, peanut HRS genes tended to cluster with genes from dicotyledons (Figure 3). The HSP90 genes from barley, wheat and rice clustered in the same clade (Figure 3). A similar result was found for RAR1 genes (Figure 6). The phylogenetic results indicated that dicot and monocot HRS genes diverged during evolution, and a similar result was found in tobacco (Song et al., 2019).

The *AhRAR-A* and *AhRAR1-B* fragments were isolated from the cultivated peanut genome. Similar to *Arabidopsis* RAR1 (Shirasu et al., 1999), the *AhRAR-A* and *AhRAR1-B* sequences each had one 3-bp exon (Supplementary Figure 2), which suggested the same splice patterns in *Arabidopsis* and peanut. This study found that the sequence of *RAR1* from cultivated peanut was quite different from that of wild peanut. *RAR1* from wild peanut consisted of two more exons. This result suggested that there were different splice patterns in the wild peanut genome or that incorrect genome assembly occurred. The *AhSGT1* genes were isolated from cultivated peanut and found that *AhSGT1-A3* and *AhSGT1-B3* are orthologous genes of the *A. diogoi SGT1* gene (Kumar and Kirti, 2015; Supplementary Figure 4), which suggested that *AhSGT1-A3* and *AhSGT1-B3* represent potential genes for improving leaf late spot disease in peanut. HSP90 usually interacts with its cochaperones RAR1 and SGT1 to perform functions (Wang et al., 2015; Situ et al., 2020). In *Arachis*, *AhRAR-A*, *AhHSP90-A10*, and *AhSGT-A3* were clustered together at the end of chromosome 9, and *AhRAR-B*, *AhHSP90-B10*, and *AhSGT-B3* were clustered together at the end of chromosome 19. The orthologous genes of AhRAR1-A and AhHSP90-A10 might be required for AdSGT1-mediated resistance to leaf late spot disease in the wild peanut *A. diogoi*. In common wheat, all HSP90 genes consist of 3 exons and 2 introns (Wang et al., 2011), while in peanut, the number of exons ranges from 3 to 20 (Figure 4). Only 9 HSP90 genes in the present study contained 3 exons. These results suggest that different evolutionary patterns of HSP90 genes exist between dicots and monocots.

### Expression Profiling of HRS Genes in Different Tissues and Stress Conditions

The expression patterns of genes can provide insights into gene functions (Zhang et al., 2013). RNA-seq is considered a powerful means to study gene expression profiles (Yuan et al., 2019). With the reduction in the cost of RNA-seq, more studies about peanut transcriptome sequencing in different tissues or under different stresses have emerged (Clevenger et al., 2016; Zhao et al., 2018, 2020; Chen et al., 2019; Zhang et al., 2020), which facilitates HRS-related gene expression pattern analysis. The expression patterns of all HRS-related genes were detected based on Clevenger et al. (2016) (Figure 7). This study found that several genes exhibited tissue-specific expression patterns and that their orthologous genes showed similar expression patterns. For example, *AdHSP90-10* and its orthologous gene *AiHSP90-10* were only expressed in the subterranean gynophore tip (SubGynTip) (Figure 7). The gynophore, which is also called the peg, is a specialized peanut organ that transitions from an upward growth habit to downward outgrowth (subterranean gynophore) upon fertilization (Chen et al., 2016). This study speculate that these two genes might play important roles in the gynophores of subterranean swelling pods. *AdHSP90-1*, *AdHSP90.6*, and *AdHSP90.8* were ubiquitously and highly expressed in all 22 tissues that were examined (Figure 7), as were their orthologous genes *AiHSP90-1*, *AiHSP90.6*, and *AiHSP90.8*. These results indicated that these genes play important roles in various physiological activities of peanut. The peanut *RAR1* gene was also expressed in all 22 tissues. The expression of *AiHSP90-6* in roots was higher than that in leaves and stems, which might indicate its involvement in sodium transport or water absorption in roots to improve peanut salt/drought resistance. As expected, its orthologous gene *AhHSP90-B6* was greatly upregulated upon salt or drought treatment (Figure 8). The promoters of those genes that are ubiquitously expressed or that show tissue-specific patterns could be potential tools for peanut quality improvement (Yuan et al., 2019).

Numerous studies have shown that HRS-related genes are involved in the responses to both biotic and abiotic stresses (Liu et al., 2006; Kadota and Shirasu, 2011; Wang et al., 2017). To reveal the responses of HRS-related genes to biotic and abiotic stresses, transcriptome sequencing data of plants under salt, drought, low calcium and *A. flavus* infection treatments were analyzed (Zhao et al., 2018, 2020; Chen et al., 2019; Zhang et al., 2020). The log2-fold changes under stress treatment are shown in a heatmap (Figure 8). This study found several HRS-related genes involved in the responses to salt, drought, low calcium and *A. flavus* infection. The expression of *AhHSP90-A5*, *AhHSP90-A9*, *AhHSP90-B3*, and *AhHSP90-B6* was significantly upregulated under both salt and drought stresses. However, RAR1 genes seemed to be insignificantly involved in stress responses. This finding implies that *Arachis* HSP90 genes participate extensively in the responses to salt and drought stress but do not require RAR1. Similarly, previous studies revealed that HSP90 genes were required for R protein-mediated disease resistance in a RAR1- and/or SGT1-independent manner (Hubert et al., 2003; Bhattarai et al., 2007). A previous study showed that *Arabidopsis HSP90.7* helped seedlings resist a high concentration of Ca^2+^ (Chong et al., 2015). In our study, we found that under low calcium conditions, *AhHSP90-B10* was upregulated, while *AhHSP90-A8* and *AhHSP90-B3* were downregulated. The HRS complex is widely involved in R gene-mediated disease resistance in plants (Shirasu, 2009; Kadota and Shirasu, 2011). Aflatoxin contamination caused by *A. flavus* is a serious problem for peanut quality (Guo et al., 2009). To reveal the responses of HRS genes to *A. flavus*, their expression profiles under *A. flavus* infection were analyzed (Zhao et al., 2020). *AhHSP90-A1*, *AhHSP90-A7*, *AhHSP90-A8* and their orthologous genes exhibited higher expression levels than other genes under infection, suggesting potential roles of these genes in combatting *A. flavus* infection. However, the R gene against *A. flav*us infection stabilized by HSP90 proteins is still unknown. In summary, HRS-related genes, especially *HSP90* genes, which are strongly involved in salt and drought stress responses, participate in both biotic and abiotic stress responses.

### Interaction Relationship Between HRS Proteins

Usually, HSP90, RAR1, and SGT1 can interact with each other, and the interactions among them are essential for their functions in plant defense (Shirasu, 2009; Pei et al., 2015). Here, HRS-related genes (including *AhRAR1-A*, *AhRAR1-B*, *AhSGT1-B1*, *AhSGT1-A2*, *AhSGT1-B2*, *AhSGT1-A3*, *AhSGT1-B3*, and *AhHSP90-B8*) from cultivated peanut were isolated to test their autoactivation and interaction in more detail using a Y2H assay. When used as prey and bait proteins, all of the detected proteins except the AhSGT1-B1 bait in TDO showed no or insignificant autoactivation on TDO and QDO media (Figure 9). A similar result was observed in barley (Pei et al., 2015), suggesting that AhSGT1-B1 cannot be used as bait for library screening. Previous studies have shown that RAR1 and SGT1 strongly interact with each other in many plant species (Wang et al., 2008, 2015; Fu et al., 2009; Pei et al., 2015). In this study, strong interactions between two AhRAR1 proteins and three AhSGT1 proteins were detected (Figure 10), which might be attributable to the interaction of the CHORD-II domain in RAR1 with the CS domain in SGT1 (Azevedo et al., 2002). AhHSP90-B8 were chose as a representative to investigate its interaction with AhRAR1 and AhSGT1. As in barley (Pei et al., 2015), RAR1 and SGT1 genes in peanut interacted with AhHSP90-B8 in low stringency medium (TDO) (Figure 10). These results indicated that AhRAR1, AhSGT1 and AhHSP90-B8 might perform functions together in peanut.

## Data Availability Statement

Publicly available datasets were analyzed in this study. This data can be found here: https://www.peanutbase.org/search/gene/.

## Author Contributions

CLY, CL, QS, and SS conceived and designed the experiments. CLY, QS, CL, XZ, CXY, and JW performed the experiments and analyzed the data. CLY, QS, and SS wrote the manuscript. All authors read and approved the final manuscript.

## Conflict of Interest

The authors declare that the research was conducted in the absence of any commercial or financial relationships that could be construed as a potential conflict of interest.

## Publisher’s Note

All claims expressed in this article are solely those of the authors and do not necessarily represent those of their affiliated organizations, or those of the publisher, the editors and the reviewers. Any product that may be evaluated in this article, or claim that may be made by its manufacturer, is not guaranteed or endorsed by the publisher.

## Footnotes

1
http://peanutbase.org/

2
https://www.ncbi.nlm.nih.gov/

3
http://smart.embl-heidelberg.de/

4
http://pfam.xfam.org/

5
http://web.expasy.org/compute_pi/

6
http://mapinspect.software.informer.com

7
http://gsds.cbi.pku.edu.cn

8
http://meme-suit.org

9
http://www.clontech.com/

## Supplementary Material

Supplementary Figure 1 Amino acid comparison between AhRAR1-A and AhRAR1-B.

Supplementary Figure 2 Gene structure of RAR1 from cultivated peanut. **(A)** Comparison of DNA sequence between AhRAR1-A, AhRAR1-B and AtRAR1. **(B)** The numbers and nucleotide acid length of exons and introns of AhRAR1-A, AhRAR1-B and AtRAR1. **(C)** Deduced amino acid sequence of RAR1. Domains CHORD-I (position 9 to 68), CCCH (100 to 118), and CHORD-II (159 to 218) are boxed. Invariant cysteine and histidine residues are indicated in red letters.

Supplementary Figure 3 Amino acid comparison among SGT1-1 genes.

Supplementary Figure 4 Amino acid comparison among SGT1-3 genes.
