# Supplementary figures and images for: Genome-Wide Identification and Characterization of HSP90-RAR1-SGT1-Complex Members From Arachis Genomes and Their Responses to Biotic and Abiotic Stresses
Source: Front Genet. 2021 Aug 27;12:689669. doi: 10.3389/fgene.2021.689669 (PMC8430224; doi:10.3389/fgene.2021.689669)

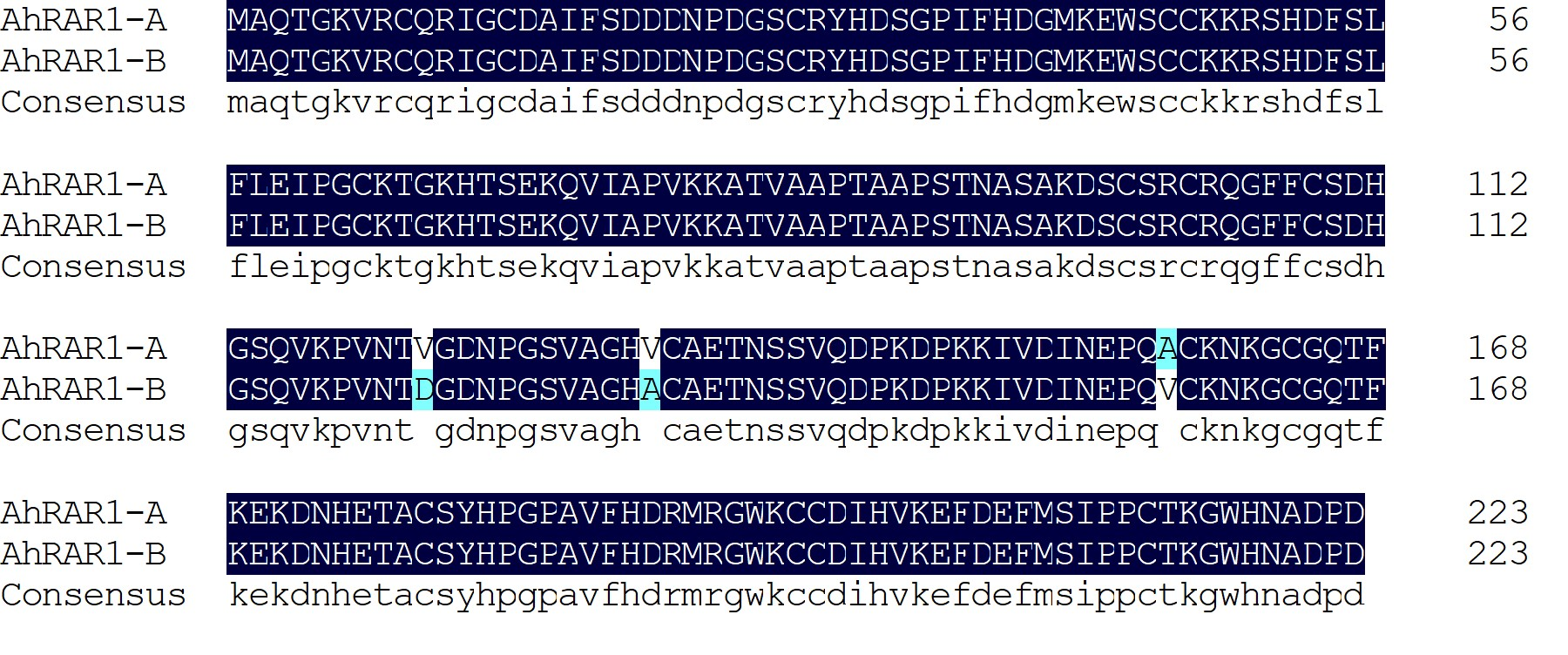

Supplement: Supplementary Figure 1 — Amino acid comparison between AhRAR1-A and AhRAR1-B. [file Image_1.TIF]

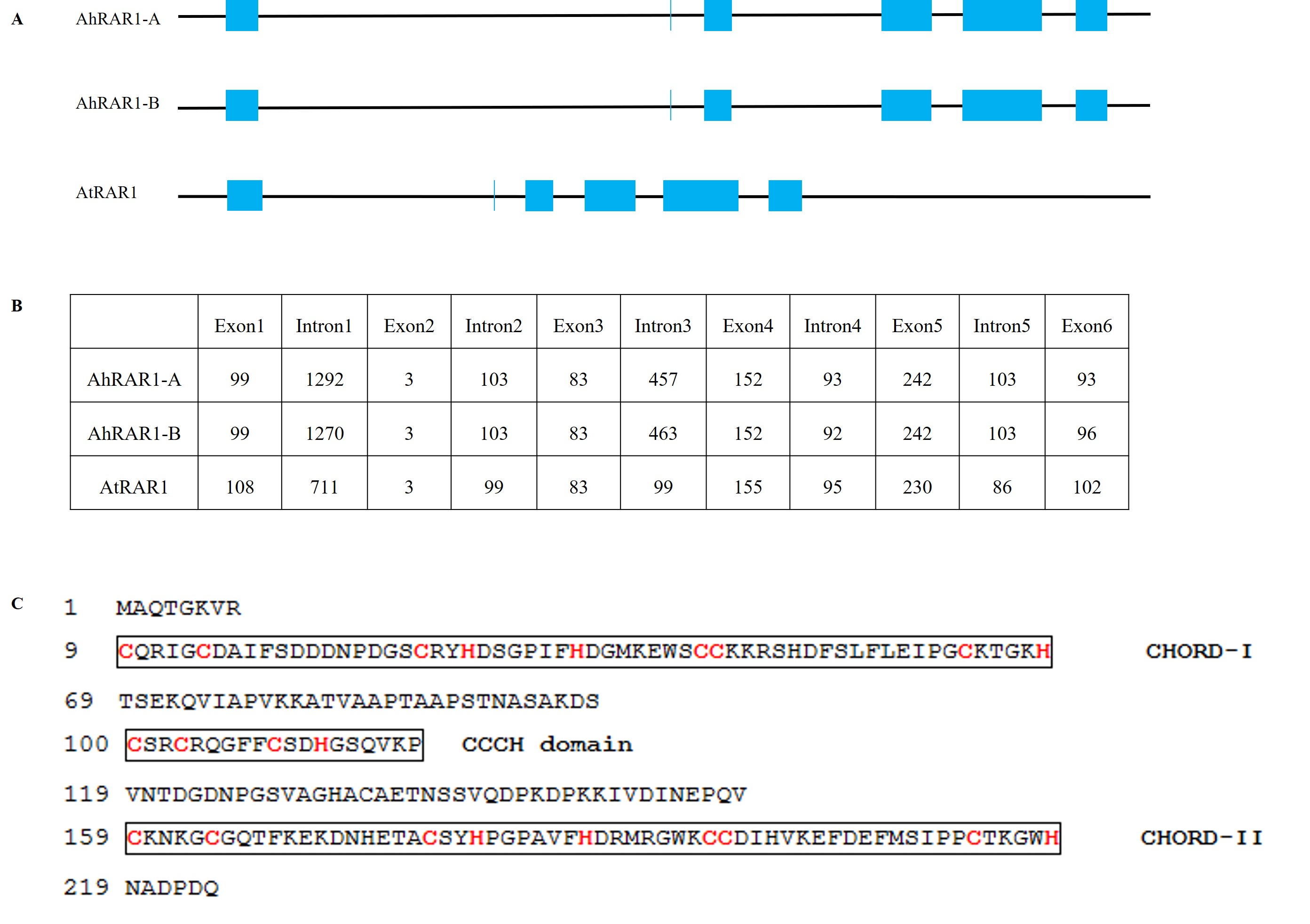

Supplement: Supplementary Figure 2 — Gene structure of RAR1 from cultivated peanut. (A) Comparison of DNA sequence between AhRAR1-A, AhRAR1-B and AtRAR1. (B) The numbers and nucleotide acid length of exons and introns of AhRAR1-A, AhRAR1-B and AtRAR1. (C) Deduced amino acid sequence of RAR1. Domains CHORD-I (position 9 to 68), CCCH (100 to 118), and CHORD-II (159 to 218) are boxed. Invariant cysteine and histidine residues are indicated in red letters. [file Image_2.TIF]

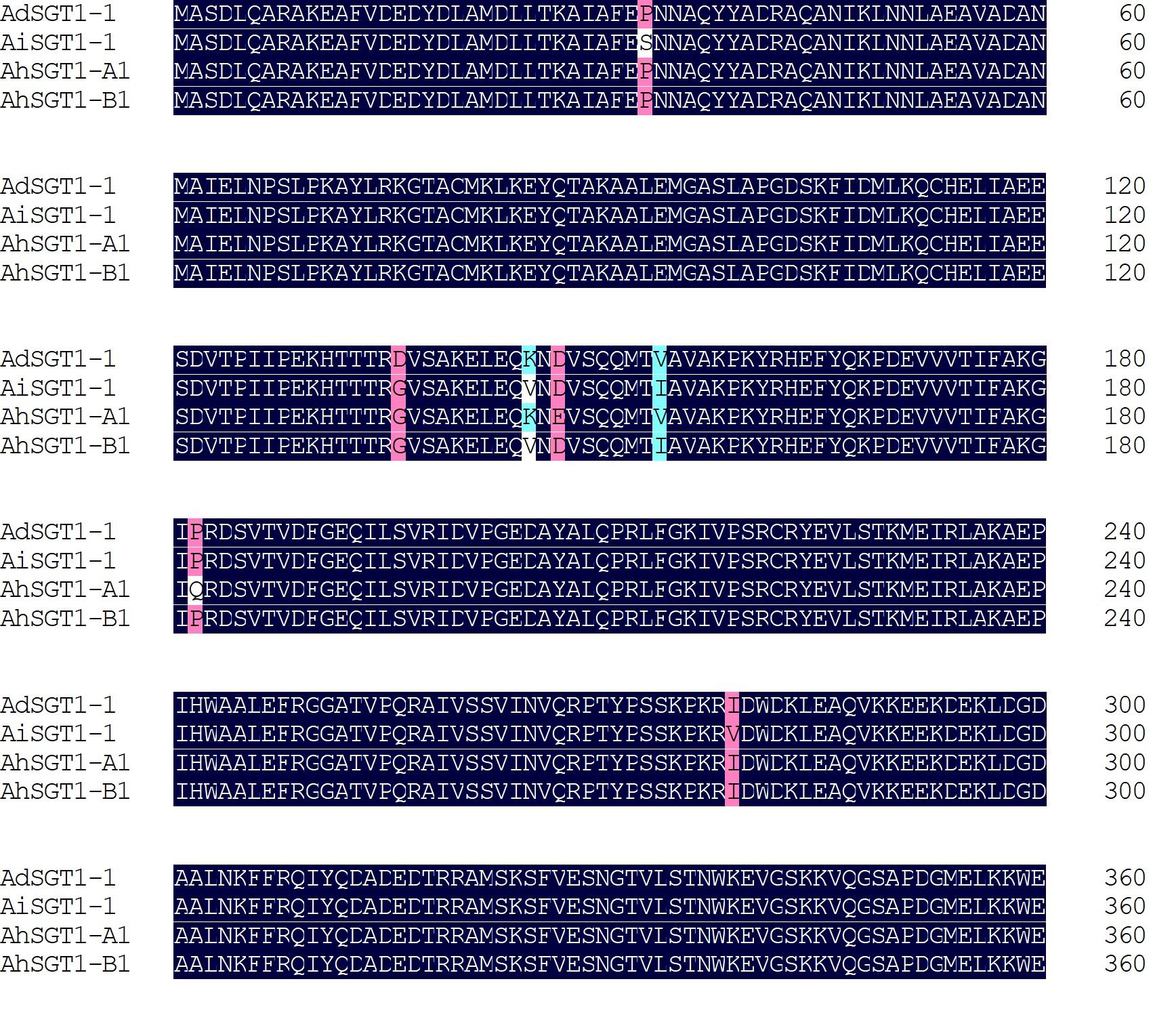

Supplement: Supplementary Figure 3 — Amino acid comparison among SGT1-1 genes. [file Image_3.TIF]

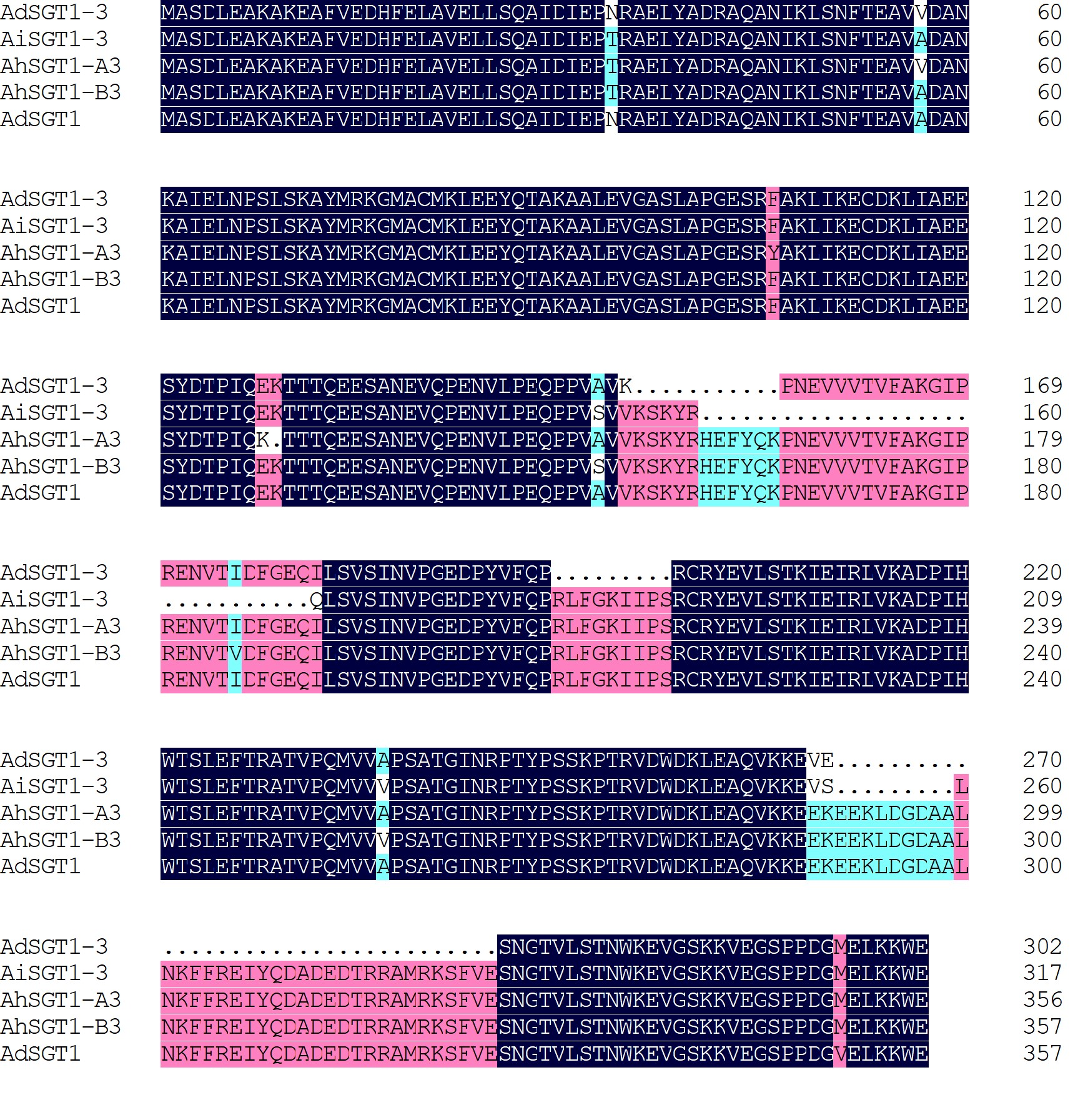

Supplement: Supplementary Figure 4 — Amino acid comparison among SGT1-3 genes. [file Image_4.TIF]
